# Novel Approach to Rule-Out Unnecessary Urine Bence Jones Protein Testing: A Serum Free Light Chain Algorithm

**DOI:** 10.3390/diagnostics15050525

**Published:** 2025-02-21

**Authors:** Vanessa García Moreira, Javier Cepeda Piorno, Jùlia Sanders Vegara, Ana Eyo González, Cristina Alberdi García del Castillo, Claudia González García, Nana Vaktangova, Sandra García Castañón, Daniel Al Kassam Martínez, Paula Chávez Collazos, Esther González García

**Affiliations:** 1 Servicio de Análisis Clínicos, Hospital Universitario de Cabueñes, 33394 Gijón, Asturias, Spain; 2 Facultad de Enfermería de Gijón, Universidad de Oviedo, 33394 Gijón, Asturias, Spain; 3 Instituto de Investigación Sanitaria del Principado de Asturias (ISPA), 33011 Oviedo, Asturias, Spain; 4 Servicio de Bioquímica Clínica, Hospital Universitario San Agustín, 33401 Avilés, Asturias, Spain; 5 Facultad de Medicina, Universidad de Oviedo, 33006 Oviedo, Asturias, Spain; 6 Servicio de Bioquímica Clínica, Hospital Universitario Miguel Servet, 50009 Zaragoza, Zaragoza, Spain; sandragcastanon@gmail.com; 7 Servicio de Hematología, Hospital Universitario de Cabueñes, 33394 Gijón, Asturias, Spain

**Keywords:** monoclonal gammopathies, multiple myeloma, Bence jones protein, serum free light chain, 24 h urine testing, screening, rule-out

## Abstract

**Background/Objectives:** Bence Jones proteins (BJPs) are monoclonal immunoglobulin free light chains (FLCs) that appear in the urine of patients with plasma cell disorders, including multiple myeloma (MM), Waldenström’s macroglobulinemia (WM), or light chain amyloidosis (AL). Their presence can provide valuable information about disease progression and treatment efficacy. These proteins are typically detected through a 24-h urine collection, as recommended by clinical guidelines. However, this method can be inconvenient for both patients and laboratory personnel due to its time-consuming nature and the potential for collection errors. We propose an algorithm based on serum FLC (sFLC) to rule out the presence of BJPs and diminish the need for urine testing. **Methods**: A retrospective data analysis of 268 serum and urine samples from 44 patients with MM was performed, and cutoffs were established to predict BJP absence: total urine protein (0.115 g/L), sFLC κ/λ ratio (>0.82 λ monoclonality and <1.99 κ monoclonality), and difference of involved–uninvolved FLC (dFLC; <11.93 mg/L). A subsequent algorithm validation was performed in 716 samples from patients who underwent the same testing in routine 2023 other laboratory activity. **Results**: The validation of these cutoffs to rule out the presence of BJP showed that, if the protocol based on the sFLC κ/λ ratio and dFLC had been applied, 42% of the urine studies would have been avoided, achieving a sensitivity of 93.9% and a false negative rate of 6.11%. **Conclusions**: We propose a laboratory work protocol that would allow for the avoidance of almost half of the 24-h urine studies based on sFLC measurement, a faster and more objective alternative to urine analysis for screening out the presence of BJP, with a good sensitivity and a low false negative rate.

## 1. Introduction

Monoclonal gammopathies (MGs) or plasma cell dyscrasias are a group of premalignant and malignant disorders characterized by the production of monoclonal immunoglobulins. MGs include the usually asymptomatic entities of monoclonal gammopathy of undetermined significance (MGUS) and smoldering multiple myeloma (SMM), which can evolve to symptomatic entities, such as solitary plasmacytoma, Waldenström’s macroglobulinemia (MW), and the more aggressive multiple myeloma (MM) and primary amyloidosis (AL) [1].

The monoclonal protein (M-protein), also known as paraprotein, produced by the tumor clone of plasma cells can be in the form of intact immunoglobulin (M-Ig), in the form of free light chains (FLCs), a combination of both, or very rarely, as free heavy chains alone.

The M-protein is identified and quantified using the combination of serum protein electrophoresis (SPE) and immunofixation (sIFE) techniques. Light chain MM accounts for 15–20% of all myeloma cases. In these patients, urine electrophoresis (UPE) and immunofixation (uIFE) have traditionally been required to detect monoclonal light chains in urine (Bence Jones Protein or BJP) [2,3].

In the past 20 years, the development of the immunochemical quantification of serum FLC (sFLC) has improved the sensitivity and accuracy of MG identification. The sFLC concentration reflects the balance between the amount being produced by plasma cells and their renal clearance. Under normal circumstances, these chains are filtered through the kidneys due to their low molecular weight, which allows them to cross the glomerular membrane, and are rapidly metabolized at the proximal tubular renal level. The kidneys can metabolize sFLC in quantities that are far in excess of their production, so in healthy individuals, FLCs are not detected in urine. But if sFLCs are very abundant, like in MGs, tubular reabsorption and catabolism capacity may be saturated and cause monoclonal FLCs to appear in the urine as BJPs [4]. It is estimated that 70–80% of MM cases present BJP at diagnosis [5,6] and it can also be detected in asymptomatic patients, as in MGUS or SMM [6,7,8]. Moreover, several studies have shown that the amount of BJP correlates with tumor burden in patients with symptomatic MM [9,10].

The presence of BJP can cause renal failure through deposits of these FLCs as amyloid fibrils, which damage the proximal tubes resulting in Fanconi syndrome, or if they are deposited in the glomerulus causing light chain deposition disease [11]. Therefore, the early detection and monitoring of FLC levels can be useful in the follow-up of patients with MGs. In fact, the presence of BJP and its concentration is a criterion of response in the progression of MM according to the International Myeloma Working Group (IMWG) [12].

SPE together with sFLC immunoassays is the most sensitive testing combination of techniques at first in the screening of MG in clinical laboratories [13]. In 2009, the IMWG recommended sFLC instead of UPE in the MM screening algorithm, but the quantification of M-protein in 24-h urine remains a recommendation in the follow-up of patients with disease that is not measurable by SPE, as a criterion for the response to treatment in both MM and AL amyloidosis [14].

The evaluation of BJP requires the collection of 24-h urine to determine the amount of protein excreted daily. Although the first morning void can be useful in the detection of BJP, consensus documents and the most representative guidelines recommend the measurement of BJP to monitor and evaluate the response to therapy in 24-h urine collection. In the laboratory, this urine sample is preanalytically processed to remove any sediment and particles that may interfere with the results, either using ultrafiltration or concentration. The urine is then subjected to UPE, a process in which the proteins present in the sample are separated according to their electrical charge and size on an agarose gel. If any M-protein is detected, it is quantified in the protein electrophoresis step. After UPE, uIFE is performed to detect and distinguish the BJP present in the sample, using specific antibodies that bind to the immunoglobulins and/or their light chains, which allows for their typing. Thus, the analysis of the banding patterns obtained in the UPE and uIFE determines the presence and quantity of BJP in the sample. This whole process is complex, time-consuming, and it always has a degree of subjectivity that is inherent to the method and the technical interpreter, as they are qualitative (uIFE) or semiquantitative (UPE) techniques [15,16]. In addition to the drawbacks at the analytical level, the detection and/or quantification of BJP also present difficulties preanalytically and discomfort for patients, such as:

- Inconvenience: 24-h urine collection can be inconvenient and cumbersome for some patients. It requires the proper collection and storage of all urine samples throughout the day, and for the patient to be attentive and engaged throughout the collection period, which may disrupt other daily responsibilities or activities [17].
- Collection errors: There is a possibility of omitting a sample or collecting it incorrectly, which can affect the accuracy of the results [18,19].
- Risk of contamination: During the urine collection process, there is a risk of contamination of the sample, and errors may occur in the results due to the presence of external contaminants.

The disadvantages of 24-h urine collection, the cost of the analytical procedure, and the moderate percentage of positive results on the initial demand encourage the use of procedures to limit requests to only those situations where urine results will provide additional data. Classically, the amount of total protein in urine was probably the only biochemical marker used to predict the presence or absence of BJP using uIFE, although its sensitivity is very low. There are even some laboratories that work following prior screening with the quantification of free or even total light chains in urine; however, these protocols do not obviate the need to collect urine samples.

In the search for a faster and more efficient method for the detection and measurement of BJP, the objective of this work is to investigate the usefulness of automated sFLC quantification as an initial step to decide when to perform 24-h urine studies, since this test is accessible in many laboratories and is generally routinely requested in most clinical visits of patients with MG. Cutoff points and an algorithm based on the quantification of sFLC are established, which can contribute to improving screening for the absence of BJP, reducing the number of UPE and uIFE necessary, and focusing the performance of these techniques at specific moments in the monitoring of the patients where the presence of BJP cannot be ruled out with a high probability.

## 2. Materials and Methods

An observational, descriptive, and retrospective study was performed by collecting data from 268 serum and urine samples corresponding to 44 patients with MM who were diagnosed and treated and who consecutively attended the hematology department of Cabueñes University Hospital between January 2016 and December 2021. In this group, 50% were men, with a mean age of 74.3 years (range 44–96).

For the validation cohort, data from 716 paired urine and serum samples obtained from 637 patients at San Agustín University Hospital between January and December 2023 were analyzed. The patients presented with a median age of 75 years (range 19–91 years) and 59% of them were male. Patients were from hematology 84.5%, internal medicine 5.01%, nephrology 4.86%, and other departments 5.63%.

All the samples underwent UPE, uIFE, and the quantification of total protein in 24-h urine, as well as the measurement of sFLC. Specifically, in the original cohort, UPE and uIFE were performed using a semi-automated Hydrasis 2 agarose electrophoresis system (Sebia; Evry, France) following the Hydragel HR and Hydragel 2/4 IF BJ protocols, respectively. In the validation cohort, UPE was analyzed using capillary electrophoresis (CZE) on a V8-Nexus system (Helena Biosciences, Gateshead, UK) and uIFE was analyzed using agarose electrophoresis on SAS-1/SAS-2 systems (Helena Biosciences, Gateshead, UK). In both cases, the quantification of sFLCs was performed using immunoturbidimetry with the Freelite^®^ reagent (The Binding Site, part of Thermo Fisher Scientific; Birmingham, UK) on an Optilite^®^ analyzer (The Binding Site, part of Thermo Fisher Scientific; Birmingham, UK).

All the information was collected in an Excel spreadsheet (Microsoft Office Professional Plus 2016) and a statistical analysis was performed using the MedCalc Statistical Software v18.2.1 (MedCalc; New York, NY, USA). Receiver operating characteristic (ROC) curves were used to calculate the cutoff points with the highest diagnostic ability to discard the presence of BJP in urine. We constructed 2 × 2 tables with the different cutoff points for each of the markers to analyze the threshold that best confirms or rules out the presence of BJP, using the results obtained in 24-h uIFE as a method of certainty. A priori, we consider that the choice of screening strategy should be established with tests that are capable of providing a detection rate or sensitivity (S) of at least 85%, with a false negative rate (FN) of less than 5%. The study was approved by the Research Ethics Committee of our hospital.

## 3. Results

Of the 44 patients with MM studied, 50% were male (*n* = 22). The age ranged from 44 to 96 years, with a mean of 74.3 ± 11.1. Table 1 shows demographic characteristics and mean values of the biochemical parameters of the patients.

**Figure 1:**
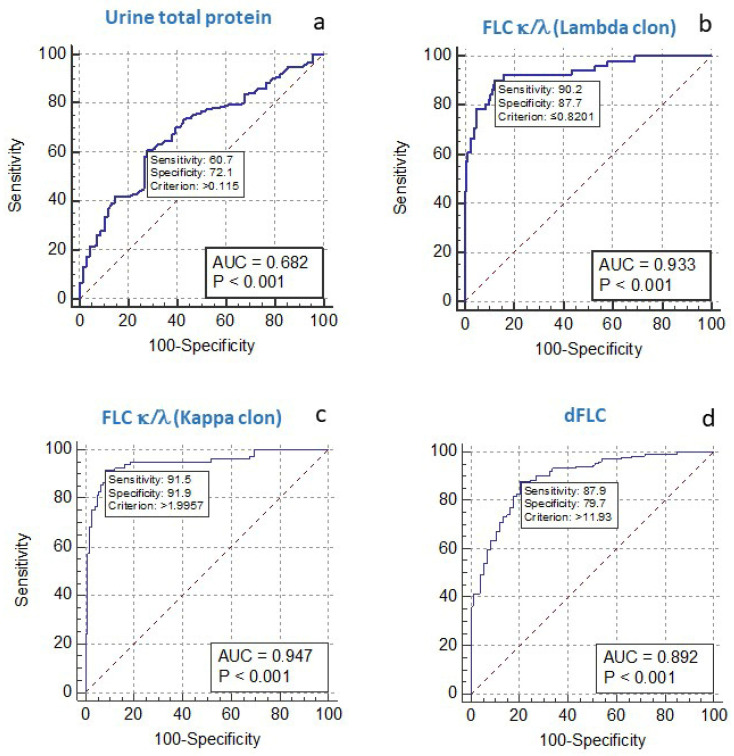
ROC curves to identify the predictive cutoffs for the presence of BJP: urine total protein values (**a**), ratio (sFLC κ/λ) (**b**,**c**), and serum free light chains difference (dFLC) (**d**). (dFLC = involved FLC—uninvolved FLC).

First, a ROC curve analysis was used to establish the cutoff point for the urine total protein concentration and sFLC that best predicts the presence of BJP. For urine proteinuria, the cutoff was found to be a concentration of 0.115 g/L, with an area under the curve (AUC) of 0.682, and an S, specificity (E) and negative predictive value (NPV) of 60.7%, 72.1%, and 50.0%, respectively (Figure 1a). As expected, the S and NPV values were not sufficiently high to consider the use of this marker as a screening test that would obviate the need for uIFE, since a high number of cases would be missed. The concordance between the absence of the monoclonal component in 24 h urine and total proteinuria <0.115 g/L was low, with a Kappa index of 0.286 (Table 2).

In the case of sFLC, since the κ/λ ratio indicates a pathological process both when the ratio is abnormally high (indicating a κ FLC producing clone) and when the ratio is abnormally low (indicating a λ FLC producing clone), it is not possible to use a single cutoff that can be useful for BJP screening. Therefore, two ROC curves were constructed to calculate the best cutoff for the sFLC κ/λ ratio in the detection of λ-type and κ-type M-proteins (<0.82 and >1.99 were used as these cutoffs, respectively) (Figure 1b,c). Given that the most clinically extended reference interval for the sFLC κ/λ ratio using the Freelite^®^ reagent is <0.26 for detecting a λ clonality, and >1.65 for detecting a κ clonality, we studied in all cases the concordance of pathological concentrations below and above the reference ranges, with the presence or absence of BJP in 24 h urine using uIFE. As shown in Table 2, the detection capacity of both sFLC κ/λ ratios improves that of the urine protein concentration, obtaining even better results with the sFLC ratio established in our laboratory, in the screening for BJP Lambda (BJL) and BJP Kappa (BJK). Thus, the cutoff of 0.82 achieves an AUC of 0.933 and S, E, NPV, and FN rates of 90.2%, 87.7%, 96.5%, and 2.42%, respectively for detecting BJL, even improving the results obtained with the traditional cutoff of 0.26. Similarly, the new sFLC cutoff calculated to detect a BJK of >1.99 achieves a better performance than the traditional 1.65, with an AUC of 0.947 and S, E, NPV, and FN rates of 91.5%, 91.9%, 94.2%, and 3.39%, respectively.

Finally, we studied the usefulness of the difference between the involved and uninvolved FLC (dFLC) in the screening for the presence of BJP to further simplify the protocol and avoid the need to use two different cutoff points in sFLC, which would also need to be adjusted to the individual characteristics of each diagnostic center in terms of the reagent and equipment used. Currently, dFLC is recognized as a very significant endpoint in AL amyloidosis and in the monitoring of oligosecretory MM. For this purpose, we constructed a ROC curve with absolute values for the dFLC concentrations with respect to the presence of M-protein in urine using uIFE (Figure 1d). In this case, the observed AUC was 0.892, which already gives an indication that the diagnostic performance of this marker is better than that of proteinuria (AUC = 0.682). The cutoff that achieved the best detection capacity was >11.93 mg/L, S = 87.9%, E = 79.7%, NPV = 78.4%, and FN and FP rates = 7.7%.

**Figure 2:**
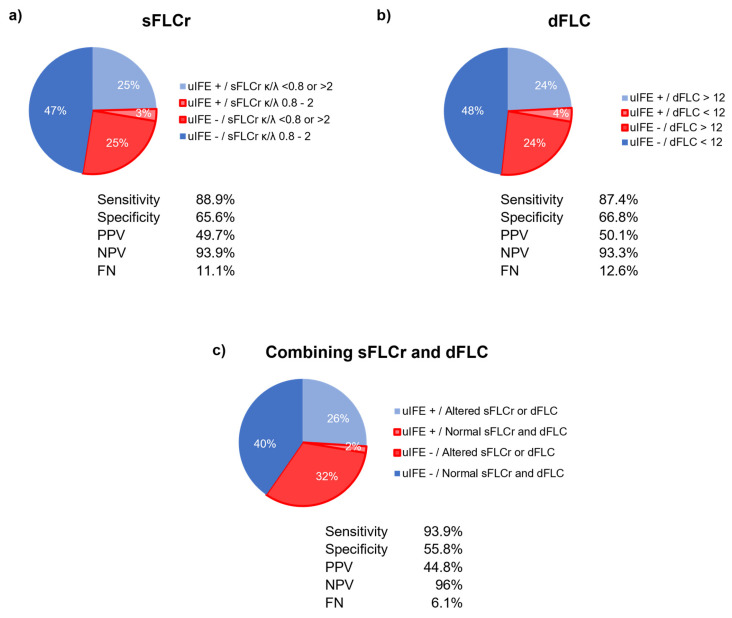
Concordances and discordances of uIFE with sFLCr (**a**), dFLC (**b**), and the combination of both parameters (**c**) in the validation cohort. PPV: positive predictive value; NPV: negative predictive value; FN: false negative.

These results were validated in a cohort of 716 paired serum and urine samples. In this sense, in the validation of the parameters and cutoffs studied, the sFLC ratio of 0.8–2 to exclude the presence of M-protein in urine using immunofixation presented S = 88.9%, E = 65.6%, PPV = 49.7%, and NPV = 93.9%, with FN = 11.1% (Figure 2a). Moreover, the dFLC < 12 obtained S = 87.4%, E = 66.8%, PPV = 50.1%, and NPV = 93.3%, with FN = 12.6% (Figure 2b).

Interestingly, the combination of both markers, thus, a sFLC ratio of 0.8–2 and dFLC < 12, is the strategy with the best diagnostic sensitivity, with NPV to be used as a screening test. Indeed, 96% (289/301) of patients with a sFLC ratio κ/λ of 0.8–2 and dFLC < 12 do not have PBJ. As shown in Figure 2c, the combination of both parameters performed with S = 93.9%, E = 55.8%, PPV = 44.8%, NPV = 96%, and FN = 6.11%.

**Figure 3:**
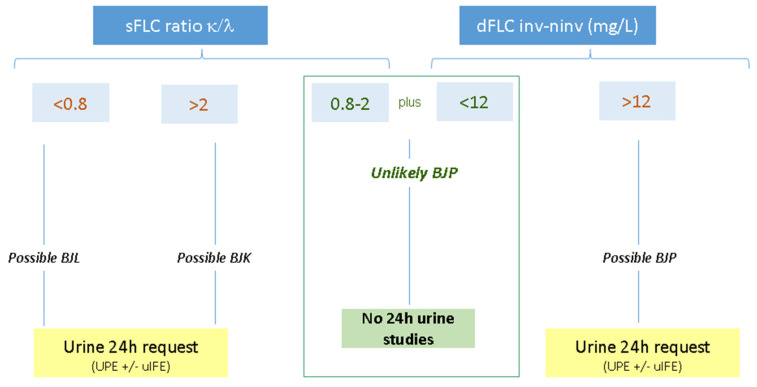
Proposed algorithm for BJP screening based on sFLC measured in an Optilite system. BJP: Bence Jones protein; BJL: Bence Jones Lambda; BJK: Bence Jones Kappa; UPE: urine proteinogram; uIFE: urine immunofixation.

Based on these results, the working algorithm represented in Figure 3 is proposed.

Altogether, the application of this algorithm in the validation cohort would have made it possible to avoid 42% of the urine tests initially requested, which can be translated into a saving of extra time (800 h) and 6600 euros in reagent costs.

## 4. Discussion

Due to its high sensitivity, uIFE is the gold standard method for detecting and typing BJP using monoclonal antibodies against the different immunoglobulin chains [20]. UPE quantifies BJP by measuring the urinary M-protein size, and providing information on tumor burden and treatment response in proliferative disorders. For a comprehensive BJP analysis, it is recommended to collect 24-h urine samples and quantify total proteinuria and BJP using both UPE and uIFE. Many laboratories initially screen with UPE, proceeding to uIFE only for samples potentially containing M-protein. However, UPE has limitations, including poor sensitivity for low protein levels and difficulty in interpretation with severe proteinuria. uIFE methods, while more sensitive, are costly and time-consuming. They require sample grouping for agarose plate preparation and rely heavily on the observer’s expertise for interpretation. So, BJP analysis involves multiple manual steps, making it expensive and lengthy despite semi-automated equipment, and it requires specially trained personnel who are experienced in protein laboratory procedures and results interpretation. Moreover, while early morning urine specimens can help to detect BJP, experts and official guidelines recommend 24-h collections for monitoring BJP levels and evaluating treatment efficacy, which can be inconvenient for patients and are prone to pre-analytical collection errors.

Among the limitations of 24-h urine studies is the fact that the detection and quantification of BJP is affected by renal metabolism [21,22]. Indeed, concentrations of sFLC κ greater than 133 mg/L and λ greater than 278 mg/L are required to exceed the proximal renal tubular reabsorption capacity and M-protein appears in urine as BJP [4]. This is a problem, since up to 50% of patients with MM have renal failure at the time of diagnosis, 20% may have acute kidney injury, and up to 10% may require dialysis [23,24,25]. In addition, there are patients with very effective renal metabolism who may have undetectable BJP despite a high sFLC production. Moreover, the collection of this type of sample is cumbersome, especially for frail and elderly patients [17], which can lead to poor compliance [26,27,28,29]. And finally, the inaccuracy in the quantification of BJP is high [18,19] and UPE and uIFE techniques are complex and have a certain degree of subjectivity at the technical level [15,16]. All of this, as well as the cost of the procedure and the low percentage of positive results on demand, makes the use of screening procedures to determine when to request testing for the detection and quantification BJP desirable.

The purpose of this study was to determine whether levels of sFLC—using both the ratio and difference—can be used in combination to improve diagnostic reliability in ruling out the presence of BJP. This is particularly relevant since most laboratories can analyze sFLC in an automated manner, potentially reducing the need for a urine analysis, which involves techniques that are tedious and subjective.

A good screening marker must have optimal S and E characteristics. These two measures are critical in assessing the performance of a marker in terms of its ability to correctly identify individuals with the condition of interest and exclude those without the condition. However, it is important to keep in mind that there is a trade-off between S and E, and in some cases, it may be difficult to achieve high values for both [30]. In the context of BJP screening, a high S is crucial to avoid missing patients with the condition, thus minimizing FN. Here, FN would be those cases with the presence of urine M-protein that would not be detected according to the cutoff points established in this study, to which no additional 24-h urine studies would be performed. Similar to the results obtained in the present study, two independent groups have reported a S above 90% for sFLC determination in comparison with 24-h urine testing [28,31]. Interestingly, more reports can be found in which sFLC and 24-h urine testing are compared if the sensitivity value is lowered down to around 80% [27,32,33,34]. It should be noted that all the forementioned studies assess clonality following a 0.26–1.65 κ/λ ratio. In this sense, the most similar study to ours could be that of Schmidt-Hieltjes and colleagues, in which they set their own reference ranges for BJP screening at 0.56–1.86 and found a 5% FN [35], which is very similar to our results (κ/λ: 0.82–1.99; FN: 2.42% and 3.39%). Interestingly, if we apply the conventional reference range of 0.26–1.65 published by Katzmann et al. [36], the FN rate obtained in our initial patient cohort is higher, especially in patients with BJL (from 2.42% to 9.71%), which illustrates the general recommendation that each laboratory should establish its own reference ranges and cutoffs, since these depend directly on the population, the reagent, and the analyzer used.

Additionally, to simplify even more the use of sera instead of urine, we also analyzed the predictive capacity of dFLC. In this sense, although dFLC does not improve the predictive capacity of the κ/λ sFLC ratio, it provides similar results and would allow the use of a single cutoff point in an algorithm based on it, which on a practical level simplifies its use.

We have always referred to uIFE as the gold standard technique to define the presence or absence of BJP, which reduces the S compared with others in which they had only performed uIFE when a suspicious pattern was observed in the UPE [28,32,35] or did not even specify the laboratory method for the 24-h urine study they used [31,33]. Indeed, prior urine concentration, as in our work, increases the detection of M-protein by UPE and uIFE, thus also affecting the S and increasing the variability between studies. Indeed, our results show that urine protein quantification is the test with the lowest BJP prediction capacity. This is because, although urinary M-protein is essentially monoclonal FLC, the degree of urinary excretion can be influenced by a variety of factors, such as light chain polymerization, the patient’s renal function, the rate of renal metabolization, etc., so despite having measurable disease in the serum, it may not be accurately reflected in urine.

The total light chain assay has been reported to detect the monoclonality of light chains in serum samples when concentrations of these proteins are greater than 4 or 5 g/L [37,38]. However, measurements of total κ and λ and their ratio do not consistently agree with the results of electrophoresis or immunofixation and, therefore, it is not a recommended test for the classification or follow-up of patients with MM [37]. Due to the relatively high concentration of circulating polyclonal Igs, the quantification of monoclonal light chains may be masked, making total light chain assays less sensitive than FLC assays, especially for the detection of LCMM or AL amyloidosis. Algorithms based on the quantification of total κ and λ light chains in urine measured using immunochemical methods have been proposed [39,40,41]; however, these are susceptible to antigen excess as well as not being specific for light chain clonality, so polyclonal chains will also be measured. Besides, the urinary levels of FLCs do not reflect the serum concentration, due to the reabsorption process and/or renal impairment that may result in an inaccurate valuation of tumor burden.

More recently, Lopez et al. have proposed an algorithm based on the ratio of total immunoglobulin light chains in urine, together with SPE and sIFE in some cases, to rule out the presence of BJP in urine. Although it achieves very good S and NPV, this algorithm does not obviate the need to collect 24-h urine, with all the drawbacks that this entails [42].

The updated guidelines of the IMWG recommend the use of sFLC in combination with SPE and sIFE for the diagnosis of MG, negating the need for urine testing except for suspected AL amyloidosis [14]. However, for monitoring treatment response in patients with MM, sFLC is recommended only for those patients in whom there is no “measurable disease” using electrophoretic methods (serum M-protein <1 g/dL by SPE and urine M-protein <200 mg/24 h by UPE) and for determining the stringent complete response (sCR) [12,43,44]. In contrast to the IMWG guidelines, Dejoie et al. proposed replacing 24-h urine testing with sFLC in the evaluation of response to treatment in all groups of patients with MM and have proposed new response criteria based on those of the IMWG [45]. In line with the French group, Bradwell et al. had already proven some time ago that urine tests could overestimate the response to therapy in patients with LCMM [46]. Importantly, a recent study by Cardenas et al. studied the substitution of urine studies by sFLC in IIMM and LCMM, concluding that, similar to the French group’s studies, urine tests can be replaced by sFLC in IIMM, while in LCMM, sFLC can be used for monitoring, leaving uIFE only to confirm CR and progression [47]. Thus, it seems increasingly clear that sFLC analysis would be advisable, in addition to diagnosis and maximum response, in intermediate analyses during monitoring and response to treatment as an alternative to 24-h urine studies. Indeed, in recent years, several studies have proposed replacing urine analysis with sFLC given its greater prognostic value and the early ability to detect progression, especially in patients who secrete M-Ig (IIMM) [18,19,48,49,50]. Interestingly, some reports have shown that there is no difference in terms of survival between patients with IIMM in CR with negative uIFE and those who lacked this result [51,52].

Recently, the Spanish group led by Vidal-Pla et al. conducted an intriguing study with a similar objective to ours: reducing the number of urine tests required for patients with MG based on serum test results [53]. They established cutoff values for serum M-protein levels in conjunction with the glomerular filtration rate to exclude the presence of urinary M-protein with a high probability. The main difference from our work is that they only included newly diagnosed patients with MG, excluding those undergoing treatment or in remission, as well as patients with monoclonal light chain components or amyloidosis. These latter groups often have low serum M-protein levels and more frequently present with urinary M-protein compared with other patients.

Our algorithm follows a similar approach but could be used as a screening tool prior to urinalysis for any type of patient with serum M-protein, regardless of their kidney function. This broader applicability could be considered a limitation of our study, as we did not separate patients with altered kidney function from those with normal function—a parameter that affects both the sFLC ratio and the difference between the kappa and lambda chains. We evaluated all the patients together since our goal was to test the usefulness of a simple sFLC-based algorithm to rule out the presence of BJP in urine under the most challenging conditions, with a mix of all types of patients at various disease stages. Had we separated these groups, we likely could have significantly improved the PPV. However, our study’s objective was not to use these markers to detect BJP with a high probability, but rather to leverage their high negative predictive value, NPV, to avoid unnecessary urine studies. Separating the groups would have complicated the algorithm, potentially requiring at least two parts with different cutoff points for each marker based on the glomerular filtration rate. While this is an avenue we could explore in a second phase to further refine the ranges for each case, it was beyond the scope of our initial study.

We have not conducted a detailed analysis on how the algorithm performs in patients with oligosecretory MM or AL amyloidosis, where the detection of BJP could have greater clinical relevance, as we did not have enough patients with these conditions to form independent groups. However, the diagnostic tests with the highest sensitivity for AL amyloidosis and oligosecretory MM are the sFLC assays, as they offer the greatest analytical sensitivity for detecting small amounts of M-protein, which other tests may fail to detect.

In the case of oligosecretory MM, approximately 70–80% of patients, whose protein levels cannot be measured using other methods, can be assessed using the sFLC assay. In fact, the IMWG recommendations for identifying monoclonal immunoglobulins at presentation suggest an algorithm based solely on sFLC and SPEP, eliminating the need for urine studies. The IMWG concluded that, for the purpose of detecting all MGs (except for amyloidosis), Freelite can replace 24-h urine evaluations, as we have previously mentioned [14].

For AL amyloidosis, the combination of SPEP, sFLC, and uIFE achieves the highest sensitivity for initially detecting a new case. However, although uIFE is more sensitive than UPEP, especially for detecting small amounts of BJP protein, the reality is that many laboratories do not perform uIFE directly on all the urine samples. Instead, they use UPEP profiles as a screening tool to determine whether an M-protein might be present and, consequently, whether uIFE should be performed. Compared with the analytical sensitivity of UPEP, sFLC assays are far more sensitive. In any case, this algorithm may not be applied in patients with suspected AL amyloidosis; as per the IMWG recommendations, uIFE should be performed alongside SPEP and sFLC to rule out this condition with a high probability. In this situation, we believe that applying the cutoffs of the proposed algorithm can help to interpret uIFE in ambiguous or doubtful cases, as sFLC provides valuable objective information.

A strength of this study is that the validation cohort for this algorithm was conducted using samples analyzed in a laboratory at another hospital. This laboratory employed the same methodology for sFLC (Freelite/Optilite) but used different equipment for both UPE and uIFE. Despite these differences, the obtained values for sensitivity, negative predictive value, and false negatives were good. This demonstrates that our algorithm can be applicable regardless of the commercial brand of analyzers used for urine tests. The Freelite assay is widely used and recognized in the clinical setting for the detection and monitoring of MG, as it is part of the screening and hematological monitoring panels recommended by the IMWG. However, it is important to note that the field of clinical diagnostics is competitive and there are other companies that offer these sFLCs with different reagents. This algorithm was developed and tested using a specific reagent and platform (Freelite/Optilite), which may affect its accuracy in laboratories using different methodologies.

This study does not aim to suggest replacing the uIFE analysis for confirming the presence or absence of BJP in complete response, nor to modify the current criteria for the MM treatment response. Additionally, it does not intend to debate whether urine analyses remain useful for identifying or monitoring MG. The detection of paraproteins in urine using uIFE and their quantification using UPE remain important at specific clinical moments in the diagnosis or monitoring of these patients and are complementary studies to the sFLC analysis. We want to identify the values of a simple serum marker that is usually requested in all visits to the hematologist in patients with MG, such as sFLC, which may obviate the need for 24-h urine evaluation in some cases. This would be applicable for screening UPE in patients without MG requested by nephrologists. The great utility that we want to show with this new algorithm is to avoid the need for urine studies at times when sFLC concentrations are indicating to us that, with a high probability, the presence of BJP can be excluded, and to indicate the need for urine studies when this exclusion cannot be affirmed. The greater sensitivity of sFLC over 24-h urine measurements may provide more accurate monitoring, allowing more cost-effective treatment management decisions [54,55,56,57]. Even for laboratories that do not wish to use a serum-only algorithm for BJP screening and perform this screening using the less sensitive UPE method rather than directly by uIFE, they should consider adding the FLC ratio and difference as valuable additional information to select samples for uIFE testing.

## 5. Conclusions

In conclusion, in the present work, we provide a sensitive, rapid, and accurate new algorithm based on serum samples that allows the screening of patients in whom the likelihood of having BJP is low. This approach reduces the inconvenience and potential errors associated with urine testing and indicates the need for urine studies when this exclusion cannot be affirmed. This has the potential to decrease the frequency of urine protein screening during surveillance for many of these patients, which would reduce the patient and laboratory burden. It would allow for more serial measurements if desired, especially in patients for whom collection may be difficult or patients who have oliguria. Its application would avoid 24-h urine collection in many cases, and we believe that screening with sFLC quantification, either with the sFLC κ/λ ratio or dFLC, could represent a significant advance in the field. This method improves the patient experience and streamlines the work of diagnostic tests related to the detection of disorders associated with urinary M-protein excretion, with a reduction in time due to the efficiency of the method.

## Figures and Tables

**Table 1:** Demographic characteristics, mean, and range concentrations of the biochemical characteristics of the patients at diagnosis.

	*n* (%)	Mean (Range)
Age in years		74.3 (44–96)
Male, *n* (%)	22 (50%)	
Hemoglobin (g/dL)		12.4 (7.6–18.3)
Serum total protein (g/L)		68 (52–106)
Creatinine (mg/dL)		0.89 (0.39–3.49)
Glomerular filtration CKD-EPI (mL/min/1.73 m^2^)		82 (15–113)
Calcium (mg/dL)		9.2 (7–14.6)
Albumin (g/L)		38 (24–46)
Lactate dehydrogenase (U/L)		335 (214–836)
β2-microglobulin (mg/L)		3.16 (1.13–14.5)
Urine total protein (g/L)		0.111 (0.03–4)
uIFE positive	138 (51.5%)	
Clonality κ	83 (31%)	3340 mg/24 h (10–42,800)
Clonality λ	55 (20.5%)	4460 mg/24 h (20–65,540)
Altered sFLC ratio	171 (63.8%)	
Elevation κ/λ ratio	121 (45.1%)	146.8 (1.65–1168)
Reduced κ/λ ratio	50 (18.7%)	0.09 (0.004–0.26)

**Table 2:** Correlation and diagnostic ability of the different cutoff points of urine protein concentrations, ratio (sFLC κ/λ), and the difference (dFLC) of free light chains in serum to identify the presence of BJP.

	Urine Protein >0.115 g/L	κ/λ Ratio <0.26	κ/λ Ratio <0.82	κ/λ Ratio >1.65	κ/λ Ratio >1.99	dFLC >11.93 mg/L
CM detection	BJP	BJL	BJL	BJK	BJK	BJP
κ index	0.286	0.65	0.714	0.754	0.819	0.662
Standard error	0.066	0.06	0.05	0.05	0.04	0.054
95% CI	0.156–0.416	0.524–0.776	0.608–0.819	0.665–0.844	0.739–0.898	0.555–0.769
AUC	0.682	0.791	0.9330	0.889	0.947	0.892
Sensitivity (%)	60.70	60.78	90.20	93.90	91.46	87.90
Specificity (%)	72.10	97.42	87.74	83.87	91.90	79.70
PPV (%)	78.70	88.57	70.76	79.38	87.21	87.88
NPV (%)	50	88.30	96.45	95.41	94.17	78.38
FP (%)	9.70	1.94	9.22	9.70	5.33	7.77
FN (%)	23	9.71	2.42	2.42	3.39	7.76

BJP: Bence Jones protein; BJL: Bence Jones Lambda; BJK: Bence Jones Kappa; AUC: area under the curve; PPV: positive predictive value; NPV: negative predictive value; FP: false positive; FN: false negative.

## Data Availability

The data that support the findings of this study are available on request from the corresponding author.
